# (*E*)-2-[4-(Piperidin-1-yl)benzyl­idene]-2,3-dihydro-1*H*-inden-1-one

**DOI:** 10.1107/S1600536810041723

**Published:** 2010-10-20

**Authors:** Mohamed Ashraf Ali, Rusli Ismail, Tan Soo Choon, Mohd Mustaqim Rosli, Hoong-Kun Fun

**Affiliations:** a Institute for Research in Molecular Medicine, Universiti Sains Malaysia, 11800 USM, Penang, Malaysia; b X-ray Crystallography Unit, School of Physics, Universiti Sains Malaysia, 11800 USM, Penang, Malaysia

## Abstract

In the title compound, C_21_H_21_NO, the indene ring system is essentially planar with a maximum deviation of 0.066 (1) Å and makes dihedral angles of 7.93 (6) and 2.43 (6)°, respectively, with the benzene plane and the mean plane of the piperidine ring. These latter two planes make a dihedral angle of 7.61 (7)°. In the crystal, mol­ecules are linked by C—H⋯O inter­actions, forming infinite chains along the *b* axis.

## Related literature

For the biological activity of chalcones, see: Di Carlo *et al.* (1999 ▶). For background to prostate cancer, see: Heidenreich *et al.* (2008 ▶); Syed *et al.* (2008 ▶). For the stability of the temperature controller used in the data collection, see: Cosier & Glazer (1986 ▶).
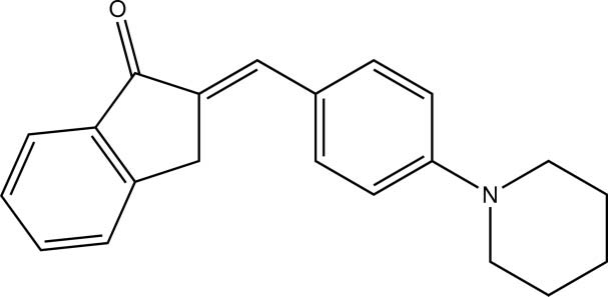


## Experimental

### 

#### Crystal data

C_21_H_21_NO
*M* *_r_* = 303.39
Orthorhombic, 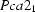

*a* = 31.587 (5) Å
*b* = 6.3168 (10) Å
*c* = 7.8396 (12) Å
*V* = 1564.2 (4) Å^3^
*Z* = 4
Mo *K*α radiation
μ = 0.08 mm^−1^
*T* = 100 K
0.48 × 0.44 × 0.09 mm

#### Data collection

Bruker SMART APEXII CCD area-detector diffractometer
Absorption correction: multi-scan (*SADABS*; Bruker, 2009 ▶) *T*_min_ = 0.963, *T*_max_ = 0.993
9828 measured reflections
2764 independent reflections
2563 reflections with *I* > 2σ(*I*)
*R*_int_ = 0.031

#### Refinement

*R*[*F* ^2^ > 2σ(*F* ^2^)] = 0.038
*wR*(*F* ^2^) = 0.103
*S* = 1.05
2764 reflections
208 parameters
1 restraint
H-atom parameters constrained
Δρ_max_ = 0.33 e Å^−3^
Δρ_min_ = −0.19 e Å^−3^

### 

Data collection: *APEX2* (Bruker, 2009 ▶); cell refinement: *SAINT* (Bruker, 2009 ▶); data reduction: *SAINT*; program(s) used to solve structure: *SHELXTL* (Sheldrick, 2008 ▶); program(s) used to refine structure: *SHELXTL*; molecular graphics: *SHELXTL*; software used to prepare material for publication: *SHELXTL* and *PLATON* (Spek, 2009 ▶).

## Supplementary Material

Crystal structure: contains datablocks global, I. DOI: 10.1107/S1600536810041723/is2615sup1.cif

Structure factors: contains datablocks I. DOI: 10.1107/S1600536810041723/is2615Isup2.hkl

Additional supplementary materials:  crystallographic information; 3D view; checkCIF report

## Figures and Tables

**Table 1:** Hydrogen-bond geometry (Å, °)

*D*—H⋯*A*	*D*—H	H⋯*A*	*D*⋯*A*	*D*—H⋯*A*
C8—H8*A*⋯O1^i^	0.97	2.44	3.3256 (18)	152

Symmetry code: (i) 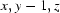.

**Fig. 1.**
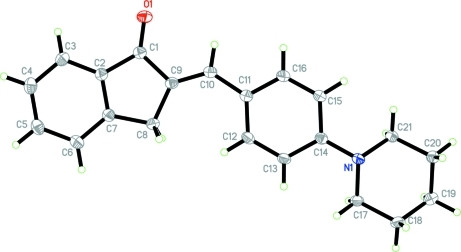
The molecular structure, showing 50% probability displacement ellipsoids and the atom-numbering scheme. Hydrogen atoms are shown as spheres of arbitrary radius.

**Fig. 2.**
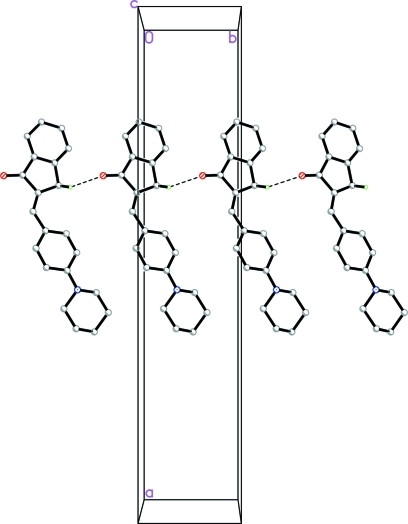
The crystal packing of (I) viewed along the b axis. Dashed lines indicate hydrogen bonds. H atoms not involved in the hydrogen bond interactions have been omitted for clarity.

## supplementary crystallographic information

### Comment

Chalcones are well known intermediates for synthesizing various heterocyclic compounds. The compounds with the backbone of chalcones have been reported to possess various biological activities. The presence of a reactive unsaturated keto function in chalcones is found to be responsible for their various biological activities. Chalcone derivatives are very versatile as physiologically active compounds and substrates for the evaluation of various organic syntheses. Chalcones belong to one of the major classes of natural products with widespread distribution in spices, tea, beer, fruits and vegetables. Chalcones also have been recently the subject of great interests for their pharmacological activities (Di Carlo *et al.*, 1999). Prostate cancer is one of the most commonly diagnosed cancers in men, and the second leading cause of cancer deaths in the European Union and United States of America (Heidenreich *et al.*, 2008). Many antitumor drugs have been developed for prostate cancer patients, but their intolerable systemic toxicity often limits their clinical use. Chemoprevention is one of the most promising approaches in prostate cancer research, in which natural or synthetic agents are used to prevent this malignant disease (Heidenreich *et al.*, 2008; Syed *et al.*, 2008).

All parameters in the title compound, (I), are within normal ranges. The indene group is  planar with the maximum deviation of 0.066 (1) Å for atom C9 and make dihedral angle of 7.93 (6) and 2.43 (6)° respectively with the C11-C16 benzene and N1/C17-C21 piperidine rings. The dihedral angle between the C11-C16 benzene ring and N1/C17-C21 piperidine ring is 7.61 (7)°.

In the crystal structure, the molecules are linked by C8—H8A···O1^i^ (Table 1) interactions to form infinite chains along the b-axis.

### Experimental

A mixture of 2,3-dihydro-1*H*-indene-1-one (0.001 mmol) and 4-(piperidin-1-yl)benzaldehyde (0.001 mmol) were dissolved in methanol (10 mL) and  30% sodium hydroxide solution (5ml) was added and the mixture stirred for 5 hour. After completion of the reaction as evident from TLC, the mixture was poured into crushed ice then neutralized with Con HCl.  The precipitated solid was filtered, washed with water and recrystallised from ethanol to reveal the title compound as light yellow crystals.

### Refinement

All H atoms were positioned geometrically and refined using a riding model [C—H = 0.93 Å for C*sp*^2^ and 0.97 Å for methine C]; *U*_iso_(H) = 1.2*U*_eq_(C) for all H atoms. In the absence of significant anomalous dispersion,1848 Friedel pairs were merged in the final refinement.

### Crystal data

**Table:**

C_21_H_21_NO	*F*(000) = 648
*M**_r_* = 303.39	*D*_x_ = 1.288 Mg m^−^^3^
Orthorhombic, *P**c**a*2_1_	Mo *K*α radiation, λ = 0.71073 Å
Hall symbol: P 2c -2ac	Cell parameters from 3558 reflections
*a* = 31.587 (5) Å	θ = 2.9–31.5°
*b* = 6.3168 (10) Å	µ = 0.08 mm^−^^1^
*c* = 7.8396 (12) Å	*T* = 100 K
*V* = 1564.2 (4) Å^3^	Plate, yellow
*Z* = 4	0.48 × 0.44 × 0.09 mm

### Data collection

**Table:**

Bruker SMART APEXII CCD area-detector diffractometer	2764 independent reflections
Radiation source: fine-focus sealed tube	2563 reflections with *I* > 2σ(*I*)
graphite	*R*_int_ = 0.031
φ and ω scans	θ_max_ = 31.5°, θ_min_ = 2.9°
Absorption correction: multi-scan (*SADABS*; Bruker, 2009)	*h* = −40→46
*T*_min_ = 0.963, *T*_max_ = 0.993	*k* = −9→9
9828 measured reflections	*l* = −11→11

### Refinement

**Table:**

Refinement on *F*^2^	Primary atom site location: structure-invariant direct methods
Least-squares matrix: full	Secondary atom site location: difference Fourier map
*R*[*F*^2^ > 2σ(*F*^2^)] = 0.038	Hydrogen site location: inferred from neighbouring sites
*wR*(*F*^2^) = 0.103	H-atom parameters constrained
*S* = 1.05	*w* = 1/[σ^2^(*F*_o_^2^) + (0.0661*P*)^2^ + 0.0999*P*] where *P* = (*F*_o_^2^ + 2*F*_c_^2^)/3
2764 reflections	(Δ/σ)_max_ < 0.001
208 parameters	Δρ_max_ = 0.33 e Å^−^^3^
1 restraint	Δρ_min_ = −0.19 e Å^−^^3^

### Special details

**Table:**

Experimental. The crystal was placed in the cold stream of an Oxford Cyrosystems Cobra open-flow nitrogen cryostat (Cosier & Glazer, 1986) operating at 100.0 (1) K.
Geometry. All esds (except the esd in the dihedral angle between two l.s. planes) are estimated using the full covariance matrix. The cell esds are taken into account individually in the estimation of esds in distances, angles and torsion angles; correlations between esds in cell parameters are only used when they are defined by crystal symmetry. An approximate (isotropic) treatment of cell esds is used for estimating esds involving l.s. planes.
Refinement. Refinement of F^2^ against ALL reflections. The weighted R-factor wR and goodness of fit S are based on F^2^, conventional R-factors R are based on F, with F set to zero for negative F^2^. The threshold expression of F^2^ > 2sigma(F^2^) is used only for calculating R-factors(gt) etc. and is not relevant to the choice of reflections for refinement. R-factors based on F^2^ are statistically about twice as large as those based on F, and R- factors based on ALL data will be even larger.

### Fractional atomic coordinates and isotropic or equivalent isotropic displacement parameters (Å^2^)

**Table:**

	*x*	*y*	*z*	*U*_iso_*/*U*_eq_	
O1	−0.17945 (3)	1.37433 (17)	0.61883 (16)	0.0241 (2)	
N1	0.04981 (4)	0.63258 (19)	0.61569 (17)	0.0179 (2)	
C1	−0.18171 (4)	1.1957 (2)	0.55722 (18)	0.0184 (3)	
C2	−0.22001 (4)	1.0902 (2)	0.48828 (18)	0.0179 (3)	
C3	−0.26187 (5)	1.1607 (3)	0.4895 (2)	0.0218 (3)	
H3A	−0.2689	1.2932	0.5326	0.026*	
C4	−0.29267 (5)	1.0262 (3)	0.4242 (2)	0.0241 (3)	
H4A	−0.3209	1.0680	0.4255	0.029*	
C5	−0.28173 (5)	0.8288 (3)	0.3566 (2)	0.0231 (3)	
H5A	−0.3028	0.7425	0.3112	0.028*	
C6	−0.23996 (4)	0.7590 (3)	0.3560 (2)	0.0217 (3)	
H6A	−0.2329	0.6274	0.3112	0.026*	
C7	−0.20910 (4)	0.8912 (2)	0.42409 (19)	0.0184 (3)	
C8	−0.16237 (5)	0.8473 (2)	0.4457 (2)	0.0200 (3)	
H8A	−0.1578	0.7202	0.5126	0.024*	
H8B	−0.1486	0.8312	0.3360	0.024*	
C9	−0.14622 (4)	1.0408 (2)	0.53840 (18)	0.0176 (3)	
C10	−0.10770 (4)	1.0829 (2)	0.60425 (19)	0.0186 (3)	
H10A	−0.1055	1.2137	0.6579	0.022*	
C11	−0.06892 (4)	0.9572 (2)	0.60519 (18)	0.0175 (3)	
C12	−0.06350 (5)	0.7587 (2)	0.52658 (19)	0.0196 (3)	
H12A	−0.0865	0.6964	0.4721	0.023*	
C13	−0.02498 (5)	0.6529 (2)	0.52761 (19)	0.0191 (3)	
H13A	−0.0227	0.5234	0.4719	0.023*	
C14	0.01083 (4)	0.7381 (2)	0.61163 (17)	0.0169 (2)	
C15	0.00507 (5)	0.9344 (2)	0.6943 (2)	0.0228 (3)	
H15A	0.0276	0.9948	0.7534	0.027*	
C16	−0.03344 (4)	1.0388 (3)	0.6890 (2)	0.0224 (3)	
H16A	−0.0358	1.1690	0.7435	0.027*	
C17	0.05603 (5)	0.4579 (2)	0.4944 (2)	0.0214 (3)	
H17A	0.0575	0.5149	0.3797	0.026*	
H17B	0.0318	0.3635	0.5000	0.026*	
C18	0.09625 (5)	0.3320 (2)	0.5307 (2)	0.0219 (3)	
H18A	0.0927	0.2543	0.6364	0.026*	
H18B	0.1006	0.2299	0.4399	0.026*	
C19	0.13536 (5)	0.4725 (2)	0.5447 (2)	0.0226 (3)	
H19A	0.1598	0.3880	0.5759	0.027*	
H19B	0.1411	0.5397	0.4358	0.027*	
C20	0.12720 (5)	0.6394 (2)	0.6797 (2)	0.0198 (3)	
H20A	0.1514	0.7338	0.6864	0.024*	
H20B	0.1239	0.5714	0.7899	0.024*	
C21	0.08766 (5)	0.7671 (2)	0.6395 (2)	0.0203 (3)	
H21A	0.0824	0.8659	0.7318	0.024*	
H21B	0.0925	0.8487	0.5365	0.024*	

### Atomic displacement parameters (Å^2^)

**Table:**

	*U*^11^	*U*^22^	*U*^33^	*U*^12^	*U*^13^	*U*^23^
O1	0.0280 (5)	0.0152 (5)	0.0289 (5)	0.0000 (4)	0.0002 (5)	−0.0022 (4)
N1	0.0192 (5)	0.0134 (5)	0.0211 (5)	−0.0015 (4)	−0.0024 (4)	−0.0024 (5)
C1	0.0204 (6)	0.0165 (6)	0.0182 (6)	−0.0006 (5)	0.0019 (5)	0.0018 (5)
C2	0.0190 (6)	0.0168 (6)	0.0179 (5)	−0.0003 (5)	0.0008 (5)	0.0010 (5)
C3	0.0215 (7)	0.0227 (7)	0.0213 (6)	0.0037 (5)	0.0003 (5)	0.0011 (6)
C4	0.0196 (6)	0.0317 (8)	0.0210 (6)	0.0000 (6)	−0.0004 (5)	0.0035 (7)
C5	0.0218 (7)	0.0283 (8)	0.0190 (6)	−0.0054 (6)	−0.0012 (5)	0.0015 (6)
C6	0.0234 (7)	0.0204 (7)	0.0212 (6)	−0.0042 (5)	0.0000 (5)	−0.0010 (6)
C7	0.0188 (6)	0.0189 (6)	0.0174 (6)	−0.0017 (5)	0.0009 (5)	0.0004 (5)
C8	0.0195 (6)	0.0171 (6)	0.0232 (6)	0.0008 (5)	0.0014 (5)	−0.0032 (5)
C9	0.0199 (6)	0.0148 (6)	0.0182 (6)	−0.0001 (5)	0.0024 (5)	0.0001 (5)
C10	0.0215 (6)	0.0165 (6)	0.0178 (6)	−0.0010 (5)	0.0018 (5)	−0.0015 (6)
C11	0.0193 (6)	0.0169 (6)	0.0163 (5)	−0.0014 (5)	0.0010 (5)	−0.0001 (5)
C12	0.0200 (6)	0.0178 (6)	0.0209 (6)	−0.0036 (5)	−0.0023 (5)	−0.0025 (5)
C13	0.0226 (6)	0.0139 (6)	0.0209 (6)	−0.0019 (5)	−0.0024 (5)	−0.0024 (5)
C14	0.0197 (6)	0.0150 (6)	0.0160 (6)	−0.0012 (5)	−0.0009 (5)	0.0004 (5)
C15	0.0203 (6)	0.0199 (6)	0.0283 (7)	−0.0004 (6)	−0.0039 (6)	−0.0079 (6)
C16	0.0210 (6)	0.0213 (7)	0.0249 (6)	0.0003 (6)	−0.0001 (6)	−0.0084 (6)
C17	0.0271 (7)	0.0149 (6)	0.0222 (6)	0.0024 (5)	−0.0059 (5)	−0.0035 (5)
C18	0.0279 (7)	0.0149 (6)	0.0230 (6)	0.0037 (5)	−0.0028 (6)	−0.0016 (6)
C19	0.0252 (7)	0.0197 (7)	0.0229 (6)	0.0033 (6)	0.0022 (5)	−0.0006 (6)
C20	0.0199 (6)	0.0173 (6)	0.0222 (6)	−0.0005 (5)	−0.0003 (5)	−0.0008 (5)
C21	0.0197 (6)	0.0147 (6)	0.0265 (7)	−0.0023 (5)	−0.0025 (5)	−0.0017 (5)

### Geometric parameters (Å, °)

**Table:**

O1—C1	1.2297 (18)	C11—C12	1.407 (2)
N1—C14	1.4004 (17)	C12—C13	1.388 (2)
N1—C17	1.4697 (19)	C12—H12A	0.9300
N1—C21	1.4785 (17)	C13—C14	1.4151 (18)
C1—C2	1.483 (2)	C13—H13A	0.9300
C1—C9	1.4951 (19)	C14—C15	1.411 (2)
C2—C3	1.395 (2)	C15—C16	1.384 (2)
C2—C7	1.397 (2)	C15—H15A	0.9300
C3—C4	1.389 (2)	C16—H16A	0.9300
C3—H3A	0.9300	C17—C18	1.525 (2)
C4—C5	1.399 (2)	C17—H17A	0.9700
C4—H4A	0.9300	C17—H17B	0.9700
C5—C6	1.391 (2)	C18—C19	1.525 (2)
C5—H5A	0.9300	C18—H18A	0.9700
C6—C7	1.390 (2)	C18—H18B	0.9700
C6—H6A	0.9300	C19—C20	1.516 (2)
C7—C8	1.512 (2)	C19—H19A	0.9700
C8—C9	1.511 (2)	C19—H19B	0.9700
C8—H8A	0.9700	C20—C21	1.520 (2)
C8—H8B	0.9700	C20—H20A	0.9700
C9—C10	1.3482 (19)	C20—H20B	0.9700
C10—C11	1.4601 (19)	C21—H21A	0.9700
C10—H10A	0.9300	C21—H21B	0.9700
C11—C16	1.3977 (19)		
			
C14—N1—C17	117.39 (12)	C12—C13—C14	121.35 (13)
C14—N1—C21	116.13 (11)	C12—C13—H13A	119.3
C17—N1—C21	113.90 (12)	C14—C13—H13A	119.3
O1—C1—C2	127.06 (13)	N1—C14—C15	121.40 (12)
O1—C1—C9	126.56 (13)	N1—C14—C13	122.18 (12)
C2—C1—C9	106.38 (12)	C15—C14—C13	116.41 (12)
C3—C2—C7	121.54 (14)	C16—C15—C14	121.22 (13)
C3—C2—C1	128.86 (14)	C16—C15—H15A	119.4
C7—C2—C1	109.53 (12)	C14—C15—H15A	119.4
C4—C3—C2	117.79 (14)	C15—C16—C11	122.87 (14)
C4—C3—H3A	121.1	C15—C16—H16A	118.6
C2—C3—H3A	121.1	C11—C16—H16A	118.6
C3—C4—C5	120.79 (14)	N1—C17—C18	112.47 (12)
C3—C4—H4A	119.6	N1—C17—H17A	109.1
C5—C4—H4A	119.6	C18—C17—H17A	109.1
C6—C5—C4	121.20 (14)	N1—C17—H17B	109.1
C6—C5—H5A	119.4	C18—C17—H17B	109.1
C4—C5—H5A	119.4	H17A—C17—H17B	107.8
C7—C6—C5	118.27 (14)	C19—C18—C17	112.63 (12)
C7—C6—H6A	120.9	C19—C18—H18A	109.1
C5—C6—H6A	120.9	C17—C18—H18A	109.1
C6—C7—C2	120.39 (14)	C19—C18—H18B	109.1
C6—C7—C8	128.13 (14)	C17—C18—H18B	109.1
C2—C7—C8	111.45 (13)	H18A—C18—H18B	107.8
C9—C8—C7	103.59 (12)	C20—C19—C18	108.49 (12)
C9—C8—H8A	111.0	C20—C19—H19A	110.0
C7—C8—H8A	111.0	C18—C19—H19A	110.0
C9—C8—H8B	111.0	C20—C19—H19B	110.0
C7—C8—H8B	111.0	C18—C19—H19B	110.0
H8A—C8—H8B	109.0	H19A—C19—H19B	108.4
C10—C9—C1	120.65 (13)	C19—C20—C21	111.33 (12)
C10—C9—C8	130.43 (13)	C19—C20—H20A	109.4
C1—C9—C8	108.88 (12)	C21—C20—H20A	109.4
C9—C10—C11	130.66 (13)	C19—C20—H20B	109.4
C9—C10—H10A	114.7	C21—C20—H20B	109.4
C11—C10—H10A	114.7	H20A—C20—H20B	108.0
C16—C11—C12	115.92 (13)	N1—C21—C20	112.69 (11)
C16—C11—C10	118.30 (13)	N1—C21—H21A	109.1
C12—C11—C10	125.77 (13)	C20—C21—H21A	109.1
C13—C12—C11	122.20 (13)	N1—C21—H21B	109.1
C13—C12—H12A	118.9	C20—C21—H21B	109.1
C11—C12—H12A	118.9	H21A—C21—H21B	107.8
			
O1—C1—C2—C3	−5.5 (3)	C9—C10—C11—C16	−177.96 (16)
C9—C1—C2—C3	174.07 (14)	C9—C10—C11—C12	2.9 (3)
O1—C1—C2—C7	177.72 (15)	C16—C11—C12—C13	−1.8 (2)
C9—C1—C2—C7	−2.73 (16)	C10—C11—C12—C13	177.35 (14)
C7—C2—C3—C4	−0.1 (2)	C11—C12—C13—C14	1.4 (2)
C1—C2—C3—C4	−176.58 (15)	C17—N1—C14—C15	−165.73 (14)
C2—C3—C4—C5	−1.2 (2)	C21—N1—C14—C15	−26.1 (2)
C3—C4—C5—C6	1.4 (2)	C17—N1—C14—C13	15.4 (2)
C4—C5—C6—C7	−0.2 (2)	C21—N1—C14—C13	155.05 (13)
C5—C6—C7—C2	−1.1 (2)	C12—C13—C14—N1	179.27 (13)
C5—C6—C7—C8	176.75 (15)	C12—C13—C14—C15	0.3 (2)
C3—C2—C7—C6	1.3 (2)	N1—C14—C15—C16	179.56 (14)
C1—C2—C7—C6	178.40 (13)	C13—C14—C15—C16	−1.5 (2)
C3—C2—C7—C8	−176.88 (14)	C14—C15—C16—C11	1.0 (3)
C1—C2—C7—C8	0.19 (17)	C12—C11—C16—C15	0.6 (2)
C6—C7—C8—C9	−175.67 (15)	C10—C11—C16—C15	−178.60 (15)
C2—C7—C8—C9	2.37 (16)	C14—N1—C17—C18	−170.28 (13)
O1—C1—C9—C10	5.7 (2)	C21—N1—C17—C18	49.17 (17)
C2—C1—C9—C10	−173.86 (13)	N1—C17—C18—C19	−52.23 (18)
O1—C1—C9—C8	−176.22 (15)	C17—C18—C19—C20	55.49 (17)
C2—C1—C9—C8	4.22 (15)	C18—C19—C20—C21	−56.53 (16)
C7—C8—C9—C10	173.83 (15)	C14—N1—C21—C20	167.89 (13)
C7—C8—C9—C1	−4.00 (15)	C17—N1—C21—C20	−51.05 (17)
C1—C9—C10—C11	178.71 (14)	C19—C20—C21—N1	55.19 (17)
C8—C9—C10—C11	1.1 (3)		

### Hydrogen-bond geometry (Å, °)

**Table:**

*D*—H···*A*	*D*—H	H···*A*	*D*···*A*	*D*—H···*A*
C8—H8A···O1^i^	0.97	2.44	3.3256 (18)	152

Symmetry codes: (i) *x*, *y*−1, *z*.
